# Screening and treatment of psychological distress in patients with metastatic colorectal cancer: study protocol of the TES trial

**DOI:** 10.1186/s12885-015-1313-y

**Published:** 2015-04-17

**Authors:** Claudia SEW Schuurhuizen, Annemarie MJ Braamse, Aartjan TF Beekman, Hanna Bomhof-Roordink, Judith E Bosmans, Pim Cuijpers, Adriaan W Hoogendoorn, Inge RHM Konings, Mecheline HM van der Linden, Elisabeth CW Neefjes, Henk MW Verheul, Joost Dekker

**Affiliations:** 1 Department of Medical Oncology, VU University Medical Center, Cancer Center Amsterdam, Amsterdam, the Netherlands; 2 Department of Psychiatry and EMGO Institute for Health and Care Research, VU University Medical Center, Amsterdam, the Netherlands; 3 VU University, Faculty of Health Science, Amsterdam, the Netherlands; 4 Department of Clinical Psychology, VU University, Amsterdam, the Netherlands; 5 Department of Medical Psychology, VU University Medical Center, Amsterdam, the Netherlands; 6 Department of Rehabilitation Medicine, VU University Medical Center, PO Box 7057, 1007MB Amsterdam, the Netherlands

**Keywords:** Colorectal cancer, Metastatic disease, Psychological distress, Screening, Quality of life, Cost-effectiveness

## Abstract

**Background/Introduction:**

Psychological distress occurs frequently in patients with cancer. Psychological distress includes mild and severe forms of both anxious and depressive mood states. Literature indicates that effective management of psychological distress seems to require targeted selection of patients (T), followed by enhanced care (E), and the application of evidence based interventions. Besides, it is hypothesized that delivering care according to the stepped care (S) approach results in an affordable program. The aim of the current study is to evaluate the (cost)-effectiveness of the TES program compared to usual care in reducing psychological distress in patients with metastatic colorectal cancer (mCRC).

**Methods:**

This study is designed as a cluster randomized trial with 2 treatment arms: TES program for screening and treatment of psychological distress versus usual care. Sixteen hospitals participate in this study, recruiting patients with mCRC. Outcomes are evaluated at the beginning of chemotherapy and after 3, 10, 24, and 48 weeks. Primary outcome is the difference in treatment effect over time in psychological distress, assessed with the Hospital Anxiety and Depression Scale. Secondary outcomes include quality of life, patient evaluation of care, recognition and management of psychological distress, and societal costs.

**Discussion:**

We created optimal conditions for an effective screening and treatment program for psychological distress in patients with mCRC. This involves targeted selection of patients, followed by enhanced and stepped care. Our approach will be thoroughly evaluated in this study. We expect that our results will contribute to the continuing debate on the (cost-) effectiveness of screening for and treatment of psychological distress in patients with cancer.

**Trial Registration:**

This trial is registered in the Netherlands Trial Register NTR4034

## Background

Colorectal cancer (CRC) is one of the most prevalent cancers and causes of cancer-related mortality in developed countries [1], with over 1.3 million new cancer cases and 694,000 deaths estimated to have occurred in 2012 worldwide in 2012 [2]. The mean 5-year survival rate is currently 59%. Approximately 40-50% of patients develop metastatic disease. Life expectancy of patients with metastatic disease is about 30 months [3].

In patients with cancer there is significant evidence of psychological distress. Psychological distress is defined as a multifactorial, unpleasant, emotional experience of a psychological (cognitive, behavioral, emotional), social and/or spiritual nature that may interfere with the ability to cope effectively with cancer, its physical symptoms, and its treatment. Distress extends along a continuum, ranging from common normal feelings of vulnerability, sadness, and fears, to problems that can become disabling, such as depression, anxiety, panic, social isolation, and existential and spiritual crisis [4]. Prior studies indicated that the majority of patients have the ability to cope with the psychological burden that can be caused by hearing the diagnosis, suffering from the disease or its treatment [5,6]. However, although precise estimates vary with different types and sites of cancer, approximately 30-40% of patients receiving cancer care experience psychological symptoms of distress, such as depression and anxiety [5,6]. These findings also apply to patients with CRC: a large proportion of patients seems to suffer from psychological morbidity [5,7]; the presence of metastases is associated with even more psychological symptoms [8,9].

Symptoms of psychological distress in patients with cancer are associated with decreased health-related quality of life and can have a large impact on the patient’s functioning [10-12]. Other studies have shown that patients with psychological distress are usually less satisfied with the care they received, show non-compliance with treatment more often [13] and use emergency room services more frequently in comparison with patients without psychological distress.

Both psychotherapeutic and pharmacotherapeutic interventions have proven to be effective in treating symptoms of psychological distress in patients with cancer [14-16]. However, a considerable number of patients are not provided with adequate psychological care, since psychological needs are often under-recognized in clinical oncology practice [10,17,18]. In order to remediate this situation, oncology guidelines have recommended to routinely screen for and treat psychological distress, thereby integrating psychological care into standard oncology practice. Nonetheless, there is lack of evidence regarding the true benefit of implementation of systemic screening and treatment of psychological distress in patients with cancer. A review of seven studies in cancer care concluded that implementation of distress screening showed limited evidence of resulting in better patient outcomes, but that treatment of identified distress itself is effective in patients with cancer [19]. Results from two recently published trials stated that the use of a psychosocial screening instrument among patients with cancer receiving radiotherapy [20] or chemotherapy [21] in itself does not sufficiently improve patients’ health-related outcome. In addition, another recent review [17] focusing on studies regarding effectiveness of screening combined with psychological treatment obtained contradictory results, but confirmed that treatment of psychological distress itself is effective in patients with cancer.

Studies conducted in both oncology and primary health care settings [21-25] indicate that merely administering screening questionnaires in clinical practice does not improve recognition, but that targeted selection of patients may result in improved recognition of psychological distress by clinicians. Targeted selection of patients involves administering and scoring of the screening instrument by someone other than the clinician, with only those with high scores offered a referral for treatment [22]. These experiences in targeted selection (or triage) seem promising in improving management of psychological distress.

Further, it has been suggested that enhanced care is needed: screening needs to be followed-up with additional assessments, follow up contacts and monitoring of the treatment process by adequately trained staff. Also extra efforts have to be made to enroll patients into appropriate referral services, where evidence-based treatments are provided (19, 20, 23–25). In addition, it has been stated that depression screening is only effective if subsequent, adequate treatment is offered. This is the conclusion from both a systematic review [23] on screening for depression in primary care, a narrative review [26] on screening for depression in various clinical settings, and a recent review on screening for psychological distress in oncology settings [27]. These findings from research conducted in patients with psychological distress combined with experiences in the area of depression lead us to hypothesize that targeted screening, enhanced care and evidence based treatment are essential ingredients of an effective approach towards screening and treatment of psychological distress in patients with cancer.

Additionally, it is important to offer psychological support in a cost-effective way, since costs are an important consideration for implementation of such a program into routine care in hospitals. In attempting to control the costs of delivering psychological interventions, the stepped care approach has been strongly advocated as being potentially cost-effective [28]. By using this strategy, intensive and costly interventions are minimalized, and reserved for those insufficiently helped by the initial interventions. Stepped care attempts to maximize the efficiency of decisions about allocation of resources in therapy, while maintaining efficacy.

We designed the TES trial, which involves Targeted selection and Enhanced care, delivered on the basis of evidence based Stepped care (TES). In this study, a screening and treatment program for distress is offered to patients with metastatic colorectal cancer (mCRC) aiming to reduce psychological distress. Our primary study objective is to evaluate the effectiveness of the TES program compared to usual care in reducing psychological distress in patients with mCRC; secondary aims include the evaluation of the impact of the TES program on quality of life, patient evaluation of care, recognition and management of psychological distress, and evaluation of the cost-effectiveness of the TES program in comparison with usual care.

## Methods

### Study design

**Figure 1:**
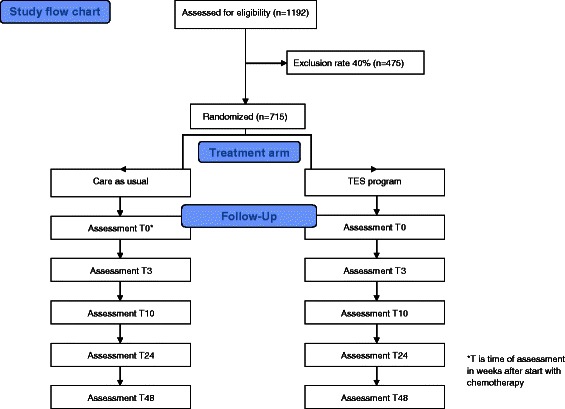
Study flow chart TES.

The study is designed as a multicenter cluster randomized controlled clinical trial with two treatment arms. Sixteen hospitals, each as a separate unit, are randomly assigned to either the TES program for screening and treatment of psychological distress *or* usual care. Psychological distress, secondary outcomes and costs are evaluated at baseline (T0), after 3 weeks (T3), 10 weeks (T10), 24 weeks (T24) and 48 weeks (T48). The design is illustrated in Figure 1.

The trial has been approved by the Medical Ethics Committee of the VU university medical center and the institutional ethics committees of the other participating sites. The study is conducted in accordance with the Declaration of Helsinki and Good Clinical Practice guidelines.

### Patients, recruitment and setting

This multicenter study is carried out in one large Dutch university medical center and 15 teaching hospitals. Patients are recruited from the outpatient clinics and inpatient wards at the Departments of Medical Oncology. Patients are eligible to participate in the study if they meet the following inclusion criteria: diagnosis of metastatic colorectal disease, start of treatment with 1st line chemotherapy, and life expectancy of more than 3 months. Exclusion criteria for participation are: age < 18 or > 85 years, insufficient command of the Dutch language, recent psychotherapy (in the past 3 months, at least one session every 2 weeks), severe psychopathology, and no informed consent.

### Randomization, treatment allocation and blinding

To allocate patients to either the TES program or to care as usual, cluster randomization is used, with hospitals as the unit of randomization to avoid contamination between groups. The randomization procedure is performed by a statistician who is blinded for hospital characteristics to ensure concealment of treatment allocation. The random allocation of hospitals is performed prior to patient recruitment. Accordingly, all participants within the same hospital are randomized into the same treatment condition (i.e. TES or usual care). Due to the nature of the intervention, neither oncologists, nurses, psychologists or their patients can be blinded. However, scoring of outcome and statistical analysis is performed blindly.

### Treatment

#### TES program in the intervention group

The key-elements of the protocol for the TES program are described below.

### Targeted selection

**Figure 2:**
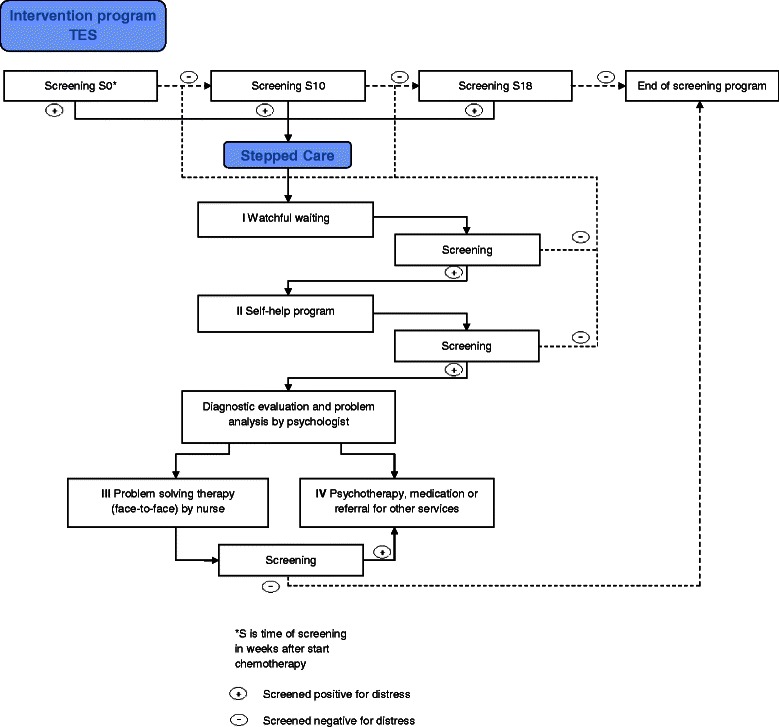
TES Intervention program.

Targeted selection is performed by a trained nurse/trained clinical nurse specialist. Screening for psychological distress is implemented for patients starting with chemotherapy, and 10 and 18 weeks later (S0, S10, and S18 – see Figure 2). The HADS (Hospital Anxiety and Depression Scale) and “Lastmeter” (Distress Thermometer and corresponding Problem List) are used as screening tools. The HADS consists of 14 questions. There are two subscales assessing anxiety and depression, which can be combined into one scale assessing psychological distress, with scores ranging from 0 to 42. The cut off score for the combined anxiety and depression scale in cancer patients is ≥ 13 [29]. The “Lastmeter” (a combination of the Distress Thermometer ranging from 0 to 10 with the Problem List and a single question on the need to talk to a professional)[30] evaluates a wide range of problems and is recommended in the Dutch guideline *“Detecting the need for psychological care”* [31]*.* In line with this Dutch guideline, and supported by various studies [30,32] we apply a cut off score of 5. The trained nurse evaluates the distress scores and offers treatment to patients who score above the cut off points on either one or both of the tools, and to patients expressing the need to talk to a professional. Furthermore, the trained nurse informs the oncologist when a patient has psychological distress or starts with stepped care.

### Enhanced care

The entire treatment process is managed by the trained nurse, who coordinates screening of all patients at the appropriate moments in time (see above); evaluates the distress scores, discusses with the patient whether he/she wants to be involved in the treatment program (regardless of the distress score) and informs the oncologist about the outcome; manages and monitors the various steps in the treatment process, as well as the transition of the patient from one step to the next step; and provides guidance for the patient during the entire treatment process.

### Stepped care

Treatment for psychological distress is delivered according to the stepped care principle [33]. The steps include: (I) watchful waiting, (II) a guided self-help program, (III) face to face problem solving treatment (PST), and (IV) psychotherapy, medication or a referral to other services (e.g. social work). Patients are screened after each step, and if psychological distress persists or if patients express the need for further guidance, a next treatment step is offered (Figure 2).

Preferably, treatment starts at step I, and then progresses to step II, III or IV, as needed. However, if the evaluation of the screening results indicates so, step I or any other step can be skipped and treatment may start at step II, III or IV. Criteria for skipping steps include major depressive disorder, family problems, severe risk of mood disturbance (risk factors include other major life events, history of psychiatric problems, young age, and little social support), the wish of the patient to skip a step, or the opinion of the oncologist that a step needs to be skipped.

Patients screening positive for distress at S10 or S18 are still allowed the full stepped care treatment.

### Step I: Watchful waiting

The first step consists of 3 weeks “watchful waiting”, as patients with psychological distress often recover spontaneously over time. This step can be chosen twice.

### Step II: Guided self-help program

If distress still persists, a guided self-help program is offered to the patient. A brief intervention for problem solving in adults experiencing common psychological symptoms such as anxiety and depression [34] has been adjusted for patients with mCRC. Both international and national randomized controlled studies have shown that problem solving treatment is effective in treating psychological distress [34-36]. Moreover, there is evidence from a growing number of trials that psychological treatments can be effectively delivered over the internet. A meta-analysis revealed that the effects of internet-based treatments for depression and anxiety disorders are comparable to those of face to face treatments [37,38]. The intervention is available as a web based version and as a booklet. The intervention takes approximately 5–7 weeks in total. In this period, patients describe what they think is important in their lives, make a list of their problems and concerns, and divide these into three categories: important and solvable problems, unimportant problems (problems that are not related to what is important in their lives); and important but unsolvable problems (such as losing someone through death). For the important and solvable problems a six-step procedure of problem solving is provided. The patient analyzes the problem and generates alternative solutions; selects and implements the chosen solution; and evaluates the results and prepares for the future. For the important but unsolvable problems, the patient receives advice and suggestions to better cope with these. Coaching is performed by the researcher or research assistant and consists of brief, weekly contacts by e-mail or telephone, which take about 10 to 15 minutes per week. The coaching is meant to give support in working through the self-help method and is not aimed at developing a patient-therapist relationship.

### Diagnostic evaluation and problem analysis

After completing step II, all patients are screened for psychological distress. In patients still meeting the criterion for psychological distress or with the desire of psychosocial support, a diagnostic evaluation and needs assessment is made by the psychologist. The results are used by the psychologist and patient to analyze problems and to evaluate further need for treatment. Together, the patient and psychologist decide on the next treatment step: face-to-face problem solving treatment (step III) or psychotherapy, medication or a referral (step IV), depending on needs and preferences of the patient. This problem analysis is performed in a maximum of 4 weeks.

In addition the Composite International Diagnostic Interview (CIDI) is used for the classification of symptoms. This is a comprehensive, fully-structured interview for the assessment of mental disorders according to the definitions and criteria of ICD-10 and DSM-IV [39] and is conducted by the research assistant by telephone.

### Step III: Problem solving treatment (face-to-face)

Problem solving treatment has shown to be effective in treating psychological distress in a wide range of somatic conditions, including cancer [40,41]. Problem solving treatment (face-to-face) is provided by the trained nurse, with the individual treatment plan serving as a guide. The treatment has a maximum of six sessions which take place in approximately 10 weeks. The treatment plan consists of identified problems, need for care, personalized goals, treatment and time for evaluation specified by the psychologist. The nurse is thoroughly trained in problem solving treatment, and follows a manual in delivering treatment [42]. If indicated, the partner is involved in one of the sessions.

### Step IV: Psychotherapy, medication or referral for other services

If psychological distress persists after step (III) or if indicated after diagnostic evaluation by the psychologist, the patient is offered psychotherapy, medication prescribed by the consultant psychiatrist, or a referral to other services (e.g. social work). Psychotherapy is delivered according to Dutch guidelines [43,44]. Suggested medication includes, among others, SSRI’s and benzodiazepines. Occasionally, antipsychotics or mood stabilizers may be needed.

### Implementation of the TES program

To create a collaborative process the medical oncologist is informed and updated about inclusion of patients in the study and treatment provided. Besides, all psychologists and nurses are thoroughly trained in the TES protocol, including the procedures for targeted selection, enhanced care and stepped care. This is done in a 1-day TES workshop. Furthermore, the approach to targeted selection, enhanced care and the various steps in the stepped care program are supervised by the psychologist from VU University medical center.

### Usual care in control group

Detection and management of psychological distress in hospitals assigned to the control group is not restricted in any way. If the patient mentions psychological distress, the oncologist interviews the patient (on an ad hoc basis, no formal screening moments for distress). During regular visits to the department of oncology, oncologists and nurses routinely ask for psychological distress and provide emotional support and advise patients on how to cope with psychological distress, on an ad hoc basis. If urgent problems emerge, the patient is referred to a psychological or psychiatric service.

### Contrast between interventions

A marked contrast between the TES program and usual care exists. Key elements of the contrast include: formal screening at regular intervals for psychological distress *versus* ad hoc interview if a patients brings up any problems or if an oncologist or nurse recognize problems; enhanced care in a collaborative team coordinated by a trained nurse *versus* regular care delivered by oncologist and nurse; diagnostic evaluation with a standardized interview assessing psychological distress and problem analysis by a psychologist *versus* non-standardized interview by oncologist and nurse; guided self-help program, individual face-to-face counseling, medication, or planned referral to other services versus ad hoc support, advice and referral to other services if needed.

### Assessment

Assessments are done in both treatment arms at T0, T3, T10, T24 and T48.

### Sociodemographic data

The following sociodemographic data are collected: age, gender, social status, employment, Dutch vs non-Dutch origin, and lifestyle characteristics.

### Medical data

Collected medical data are: diagnosis, interval since diagnosis, initial TNM stage (classification of tumors using the size and extension of the primary tumor (T), its lymphatic involvement (N), and the presence of metastases (M)), cancer treatment, comorbidity, survival, relapsed disease with concomitant treatment and death.

### Primary outcome measures

Primary outcome is the difference in treatment effect over time in reduction of psychological distress between the TES program and usual care arm. Psychological distress is measured by the Hospital Anxiety and Depression Scale (HADS). The HADS has shown to be reliable, valid, and responsive, and has been widely used in research on cancer patients (e.g. [45]). A review showed sensitivity and specificity values for the HADS of 0.8 and higher [46].

### Secondary outcome measures

Quality of life (QoL) is measured by the EORTC-QLQ-C30 version 3.0. This is an integrated system for assessing the health related quality of life of cancer patients[47]. The questionnaire incorporates five functional scales (physical, role, cognitive, emotional, and social), three symptom scales (fatigue, pain, and nausea and vomiting), a global health status / QoL scale, and a number of single items assessing additional symptoms commonly reported by cancer patients (dyspnoea, loss of appetite, insomnia, constipation and diarrhoea) and perceived financial impact of the disease. The instrument has been shown to be valid, reliable, and responsive to change [33,46].

In addition to the EORTC-QLQ-C30, the RAND-36 is used as a generic measure of health-related quality of life [48]. The RAND-36 is a generic, short-form health survey with 36 questions and consists of 8 sub-scales: physical functioning, role physical, bodily pain, general health, vitality, social functioning, role emotional, and mental health. Patients’ evaluation of care is assessed with the Client Satisfaction Questionnaire – 8 [49]. The CSQ-8 is a validated instrument to assess patient’s satisfaction with mental health care.

To evaluate recognition of and referral related to psychological distress (e.g. to psychological or psychiatric services) by clinicians in both treatment arms, patients’ medical records are reviewed by a blinded assistant. A structured form is used to extract data from the records.

### Economic evaluation

The economic evaluation is conducted from both the societal and the hospital perspective. An estimation of all relevant costs will be made in both TES and usual care: intervention costs, healthcare costs not related to the oncological treatment, informal care costs, and costs of production losses due to absenteeism from paid work. Costs of the TES intervention will be estimated using a bottom-up approach. Costs that will be included in this calculation are: 1) costs of the development of the intervention; 2) costs of training the nurses implementing the intervention; 3) costs of implementing the intervention. A detailed description of resources involved in implementing the TES intervention will be based on the logbook, prospectively completed by the nurse. Healthcare utilization outside the hospital, informal care and absenteeism from paid work will be assessed using an adapted version of the TiC-P questionnaire [50]. The adapted cost questionnaire will be administered at T10, T24, and T48 with a recall period of 3 months.

As we assume that the oncological treatment in both the experimental and control group of patients with mCRC is equal, the oncological treatment costs will not be included in the cost measurements. Absenteeism from paid work will be valued according to the friction cost method [47] using mean age- and gender-specific income of the Dutch population.

Absenteeism from unpaid work and informal care is valued using the shadow price method. The shadow price of voluntary work and informal care is assumed to be equal to the tariff for cleaning work.

Quality of life will be measured by the EuroQol (EQ-5D). Quality adjusted life years (QALY’s) will be calculated by multiplying the utility of a health state by the time spent in this health state. The Dutch valuation tariff will be used to value health states [48].

### Statistical analysis

Primary outcome is the difference in treatment effect over time in psychological distress, as assessed with the HADS, between the TES program and usual care. Secondary outcomes include quality of life, patient evaluation of care, recognition and management of psychological distress, and costs. Data are analyzed according to the intention to treat principle, using (mixed model) analysis of covariance. The exact interval between assessments is modelled in the analyses. Patients entering stepped care at S0, S10, or S18 are accounted for via a covariate in the analyses. Response to treatment (progression or not) is accounted for via a covariate as well.

In the intention to treat analysis mixed effects models for longitudinal data are used to deal with missing data (attrition). Alternatively, depending on the missing data pattern, Multiple Imputation (MI) using the Multivariate Imputation by Chained Equations (MICE) is used to impute missing effect and cost data [51-54]. The number of imputed data sets to be created is determined based on the fraction of missing information[55]. All datasets are analyzed separately and the results of the separate analyses are pooled using Rubin’s rules [56].

A complete data analysis and subgroup analysis based on patient and treatment characteristics are performed as secondary analyses. Additionally, during the study, data on the pre-study number of referrals to psychological or psychiatric services is gathered at each department of medical oncology (retrieved from medical records). If substantial differences exist, pre-study number of referrals is used as a covariate in the analyses, to correct for these differences.

### Cost-effectiveness analyses

Both cost-effectiveness analyses (using the HADS) and cost-utility analyses (using QALYs) will be performed. Missing cost and effect data are imputed using multiple imputation [54], as previously described. Differences in costs and effects are analyzed using linear multilevel regression analyses including clustering at the levels of hospitals and/or nurses implementing TES. Adjustment for confounding and/or effect modification is done if necessary. Incremental cost effectiveness ratios (ICERs) are calculated by dividing the differences in mean total costs between both treatment groups, by the difference in mean effects between both treatment groups. To account for the typically skewed distribution of costs, bias-corrected and accelerated bootstrapping (5000 replications) is used to estimate the 95% confidence intervals around the mean cost differences and the uncertainty surrounding the ICERs. To account for the clustering of data, bootstrap replications are stratified for hospital [57]. The bootstrapped ICERs are graphically presented in cost-effectiveness planes. Cost-effectiveness acceptability curves are estimated [58,59] to show the probability of the intervention program to be cost-effective in comparison with usual care for a range of different ceiling ratios, thereby showing decision uncertainty. In order to evaluate robustness of the results, sensitivity analysis on the most important cost drivers is performed.

### Sample size calculation

The study aims to evaluate whether implementation of screening and subsequent treatment is effective. The power analysis takes into account that only a subset of patients (i.e. 33%, see below) is actually treated for psychological distress; the treatment effect obtained in this subset is ‘diluted’ by patients who are not treated for psychological distress. The power analysis addresses the question on the effectiveness of screening and treatment *in all patients*: the overall expected effect size is calculated in all patients, including patients who are actually treated and patients who are not treated [6]. The overall expected effect size of the implementation of screening and subsequent treatment is calculated as follows:

i. The expected proportion of patients who are treated for psychological distress is 33% [24,60]. In patients who are treated for psychological distress, the expected effect size is d’ = 0.54 [40].
ii. The expected proportion of patients who are not treated is 67%. The expected effect size in patients who are not treated is d” = 0.
iii. Thus, the overall expected effect size in the entire sample is d = 0.18.

Setting the within subject correlation rho = 0.3, the overall expected effect size d = 0.18, alpha = 0.05 (two tail), beta = 0.80, the required sample size is 604 patients (302 per group).

Since this is a cluster randomized trial, patients within a cluster (i.e. hospital) cannot be assumed to be independent. To correct for the clustering effect, the obtained sample size should be inflated [61]. Assuming an intra cluster correlation of 0.005 [62] and a cluster size of 38 (604 divided by 16 hospitals), the obtained sample size is multiplied by 1 + 0.005 (38–1) = 1.18 [62]. This results in 604 x 1.18 = 715 patients.

Setting the exclusion rate at 40% (primarily due to lack of informed consent), 1192 eligible patients are required. Patients are recruited during 28 months in 16 oncology departments, which on average see 35 eligible patients per year. This results in an estimate of 1300 patients. Thus, recruitment is feasible, even taking into account a high exclusion rate. In previously published intervention studies on quality of life aspects, however, about 70% of eligible patients agreed to participate [24,63,64], and an exclusion rate of 40% seems rather pessimistic.

## Discussion

With the current study, we aim to evaluate the effectiveness of the TES program, using screening, enhanced care and a multi-disciplinary and stepped care oriented approach in which well-described effective psychotherapeutic and pharmacotherapeutic interventions are implemented, compared to usual care in reducing psychological distress in patients with mCRC. Since symptoms of distress are relatively common in CRC patients and the availability of professional support is limited, there is a need for evidence-based knowledge regarding effectiveness of integrated psychological care for cancer patients who need extra support. Yet, up to now, studies on screening and treatment of psychological distress in cancer patients have yielded mixed results. The most important strength of the present study is that we use targeted selection, enhanced care and effective interventions to create optimal conditions for a screening and treatment program for psychological distress in patients with mCRC to be effective. Another strength of the study is the clear distribution of tasks in a collaborative team with multiple professional disciplines brought together. With the nurse executing a coordinating role in the treatment process, the professionals have the opportunity to deliver care efficiently. In addition, the study follows the set of recommendations for randomized trials made by CONSORT [65] and in that way differentiates itself from several earlier published studies on this issue.

An innovative aspect of this trial is that the intervention aims at a balance between efforts and inputs. This is realized by choosing for a stepped care approach, in which patients start with the least intensive treatment that is most likely to work, with more intensive and costly interventions reserved for those insufficiently helped by initial steps. To our knowledge, this is the first trial in which the cost-effectiveness of screening and treatment of psychological distress in CRC patients in comparison with usual care is determined. Previous research focused on cost-effectiveness of implementing a screening programs for patients with cancer, but did not include further psychological assessments and referral to appropriate services [21].

A possible limitation might be that former studies revealed that a large number of patients with psychological problems do not actually use supportive psychological resources [66]. The main reason for non-use seemed to be patients’ belief that they do not need any help, followed by lack of knowledge about the services provided and personal attitudes towards psychosocial treatment [66]. According to another study, as many as 30% of the patients who reported symptoms of anxiety or depression declined support [67]. Since it has been found that a significant portion of this group do not take part in psychological support offered, a stepwise approach will be used in the present study to individualize the level of support needed, assuring tailored care. In such a way we hope to increase the acceptance and accessibility of psychological care by patients that really benefit from treatment for psychological distress.

Another possible limitation is the homogeneity of the study population included in the trial; results may not be transferable to patients with cancer in general. Cancer type specific characteristics, e.g. average age of onset, course of disease, and associated physical impairment, may influence patients’ level of distress and their response to supportive psychological interventions. Results obtained from this trial in patients with mCRC may therefore not equal those that would be found in another cancer type. However, to evaluate the effectiveness of the TES program we decided to restrict our study population to this specific patient group in order to prevent influences from different types of cancer on the outcome.

To conclude, the present study is designed to assess the TES program on effectiveness, with regard to psychological distress, costs, quality of life, patient evaluation of care, and recognition and management of psychological distress in patients with mCRC compared to usual care. Our results will help to resolve the continuing debate on the (cost-) effectiveness of screening for and treatment of psychological distress in patients with cancer. If the trial proves a successful outcome, the TES program can be made available for implementation on a larger scale in clinical practice.

### Hospitals participating in the TES trial

1. VU University medical center, Department of Medical Oncology, Department of Psychiatry, Amsterdam, The Netherlands
2. Amstelland Ziekenhuis, Amstelveen, The Netherlands (CJ van Groeningen)
3. Medisch Centrum Alkmaar, Alkmaar, The Netherlands (S Vrijaldenhoven)
4. Spaarneziekenhuis, Hoofddorp, The Netherlands (A Beeker)
5. Meander Medisch Centrum, Amersfoort, The Netherlands (HJ Bloemendal)
6. Waterlandziekenhuis, Purmerend, The Netherlands (J Brakenhoff)
7. Zaans Medisch Centrum, Zaandam, The Netherlands (A van Bochove)
8. Gemini Ziekenhuis, Den Helder, The Netherlands (C Tromp)
9. Rode Kruis Ziekenhuis, Beverwijk, The Netherlands (R Rietbroek)
10. Medisch Spectrum Twente, Enschede, The Netherlands (ANM Wymenga)
11. St. Antonius Ziekenhuis, Utrecht, The Netherlands (M Los)
12. Diaconessenhuis, Leiden, The Netherlands (E Batman)
13. Flevoziekenhuis, Almere, The Netherlands (V Lustig)
14. Rijnstate, Arnhem, The Netherlands (MJDL van der Vorst)
15. Medisch Centrum Leeuwarden, Leeuwarden, The Netherlands (M Polée)
16. To be determined

## Abbreviations

TES: Targeted Selection, enhanced and stepped care
CRC: Colorectal cancer
mCRC: Metastatic colorectal cancer
HADS: Hospital anxiety and depression scale
QoL: Quality of Life
CSQ: Client Satisfaction Questionnaire
QALY’s: Quality adjusted life years
ICERs: Incremental cost effectiveness ratios
